# Investigating resistance to 5-Azacytidine and Venetoclax in PDX models of MDS/AML

**DOI:** 10.3389/fonc.2024.1414950

**Published:** 2025-01-07

**Authors:** Petra Bašová, Lubomír Minařík, Silvia Carina Magalhaes-Novais, Jana Balounová, Zuzana Zemanová, Tatiana Aghová, Martin Špaček, Anna Jonášová, Kristýna Gloc Pimková, Jan Procházka, Radislav Sedláček, Tomáš Stopka

**Affiliations:** ^1^ BIOCEV, First Faculty of Medicine, Charles University, Prague, Czechia; ^2^ Department of Hematology, General Faculty Hospital and Charles University, Prague, Czechia; ^3^ Czech Centre for Phenogenomics, Institute of Molecular Genetics of the Czech Academy of Sciences, Prague, Czechia; ^4^ Department of Biochemistry and Laboratory Diagnostics, General Faculty Hospital and Charles University, Prague, Czechia

**Keywords:** myelodysplastic syndrome, PDX (patient derived xenograft), 5-Azacytidine, Venetoclax (BCL2 inhibitor), therapeutic targets

## Abstract

**Introduction:**

Progressing myelodysplastic syndrome (MDS) into acute myeloid leukemia (AML) is an indication for hypomethylating therapy (HMA, 5-Azacytidine (AZA)) and a BCL2 inhibitor (Venetoclax, VEN) for intensive chemotherapy ineligible patients. Mouse models that engraft primary AML samples may further advance VEN + AZA resistance research.

**Methods:**

We generated a set of transplantable murine PDX models from MDS/AML patients who developed resistance to VEN + AZA and compared the differences in hematopoiesis of the PDX models with primary bone marrow samples at the genetic level. PDX were created in NSGS mice via intraosseal injection of luciferase-encoding Lentivirus-infected MDS/AML primary cells from patient bone marrow. We validated the resistance of PDX-leukemia to VEN and AZA and further tested candidate agents that inhibit the growth of VEN/AZA-resistant AML.

**Results and discussion:**

Transplantable PDX models for MDS/AML arise with 31 % frequency. The lower frequency of transplantable PDX models is not related to peritransplant lethality of the graft, but rather to the loss of the ability of short-term proliferation of leukemic progenitors after 10 weeks of engraftment. There exist subtle genetic and cytological changes between primary and PDX-AML samples however, the PDX models retain therapy resistance observed in patients. Based on *in vitro* testing and *in vivo* validation in PDX models, Panobinostat and Dinaciclib are very promising candidate agents that overcome dual VEN + AZA resistance.

## Introduction

Although myelodysplastic syndrome (MDS) during progression to acute myeloid leukemia (AML) is indicated for hypomethylating therapy (HMA, 5-Azacytidine (AZA)), the addition of a BCL2 inhibitor (Venetoclax, VEN) yields significantly better responses in patients unsuitable for intensive chemotherapy. Genetic predictors of a favorable response include *IDH1/2* or *DDX41* mutations, while negative predictors include monosomy 7, *EZH2* mutations or *N/KRAS* activating mutations. Although AZA and VEN have different modes of action, most patients develop dual resistance leading to relapse with an expected survival of 2.4 months (1). Recently, cellular models of stable dual resistance to VEN + AZA have been generated without altering their proliferation properties. Resistance to VEN + AZA leads to an increase in the anti-apoptotic protein MCL1 and a decrease in the pro-apoptotic protein BAX, which has been experimentally validated as a mechanism of resistance in these models (2). However, further research has shown that only co-targeting of BCL2 and MCL1 is effective in AML cell lines with intrinsic or acquired resistance to BH3 mimetics or engineered to genetically overexpress BCL2 or BCL2A1 or downregulate BAX. Thus, it is rather the co-dependence on multiple anti-apoptotic BCL2 proteins and BAX suppression that can be considered as mechanisms of AML resistance to individual BH3 mimetics (3). Genetic screens based on CRISPR-Cas9 have successfully identified several targets in AML, specifically MARCH5, which prevents apoptosis in AML and whose repression enhanced the efficacy of BCL2 inhibitors such as VEN (4), not only in established AML cell lines, but also in PDX models, which are considered to be the most faithful to the primary human disease.

Current patient derived xenograft (PDX) models were established by Wunderlich et al. who generated a mouse strain that transgenically expresses the human cytokines: stem cell factor (SCF), GM-CSF, and IL-3 (SGM3) in a NOD/SCID background, termed NOD/LtSz-scid IL2RG-SGM3 (NSGS), which resulted in improved engraftment of AML cells from patients with various aberrations even compared to the original NSG model (5). PDX exhibit functional heterogeneity, specifically, up to twelve AML patient-derived subclones were generated, each derived from a single stem cell. These clones exhibited different transcriptional and proteomic profiles with a molecular signature of resistance (6). Interestingly, for PDX modelling of AML, reproducible human AML engraftment is mainly limited to high-risk cases. A recent study showed that the majority (85-94%) of mice were engrafted into bone marrow (BM) regardless of risk group, but serial transplantation and long-term cell culture (LTC-IC) assays revealed long-term engraftment in patients with high-risk AML but also with high leukemia-initiating cell counts. This study provided a comprehensive *in vivo* characterization of human AML in NSGS-type mice and reveals distinct intrinsic characteristics for each risk group with respect to engraftment capacity, LIC, and stromal interactions (7).

PDX models are used to test new therapies such as HDAC inhibitors (8), PPAR alpha inhibitors that specifically inhibit leukemia stem cells (9), FLT3 inhibitors (10, 11), kinase inhibitors (12), or SF3B1 inhibitors (13). In our research, we wanted to build on these previous studies and asked how efficiently NSGS-based PDX models can be created and, more importantly, whether they can be successfully used to test new compounds overcoming resistance to VEN + AZA.

## Materials and methods

### PDX acceptor mouse strain as a model to engraft and serve for testing of MDS/AML

NOD.Cg-Prkdc^scid^ Il2rg^tm1Wjl^ Tg(CMV-IL3,CSF2,KITLG)1Eav/MloySzJ is also known as NSG-SGM3. The NSG-SGM3 (NSGS) transgenic mouse expresses the human cytokines IL3, GM-CSF (CSF2) and SCF (KITLG), thus combining the properties of the highly immunodeficient NOD scid gamma (NSG, The Jackson Laboratory) mouse, which ideally promotes stable adhesion of myeloid lineages (14) and regulatory T-cell populations through cytokine expression. However, T cell engraftment may paradoxically reduce tumor cell engraftment through the so-called graft vs leukemia effect. Nevertheless, it can be concluded that these NSG-SGM3 mice are almost ideal for implantation of primary AML samples (5) than other models and are useful for immuno-oncology, immunology and other research.

### Patient samples and ethics

BM samples (representing residua of those which were used for routine diagnostics) were cryopreserved in liquid nitrogen with patients’ agreement to further analysis. Patient samples were collected in year 2023 following the written informed consent based on the Helsinki declaration and approved by the Ethics Committee of the General Hospital Prague. On 14 June 2018, the Ethics Committee of the General University Hospital discussed and approved the grant project No. 48/18 entitled Development of mouse models of hematological tumors for the purpose of cancer research and experimental preclinical treatment, which was to be carried out in the years 2019-2023. Furthermore, on 16 June 2022, the Ethics Committee approved the grant project No. 38/22 entitled Research on resistance to immunomodulatory drugs (IMiDs) - design of new therapies.

### Mouse handling and ethics

Animals were maintained under SPF conditions in individually ventilated cages with controlled temperature (22 ± 2°C) and humidity under a 12 h light/12 h dark cycle and with food and drink ad libitum. Body condition score and body weight were accessed twice a week until the end of the experiment. Mice were sacrificed when signs of disease were visible, such as: lethargy, excessive weight loss (> 20%), change in fur quality, apathy, and hind leg paralysis. The license number for the CCP projects including the ethics was entitled Study of targeted therapy *in vivo* in PDX model of chemoresistant myeloid malignancies is PDX 63/2020 later modified AVCR 253/2023 SOVII.

### Intraosseal transplantation technique

Three days before transplantation mice were fed with high caloric diet and 2% of gentamycin (G1397, Merck) was added in their drinking water. The gentamycin was kept for 7 days from the day of transplantation and changed every 3 days. On the day of transplantation mice were irradiated with sublethal dose of 120 cGy using XRAD320 X-ray system.

For the intraosseal cell application, after anesthetized with 20% Zoletil (Biopharm), mice hind limbs were shaved and cleaned with scrubs of betadine. Mice were placed in a supine position on a sterile surgical platform and hind limbs were fixed at 90 degrees from the platform. Using 1 ml syringe with 27 gauge needle the femur was slowly perfused with 20 µl of cell suspension. Aspiration was performed to confirm proper needle placement. Post-operative analgesics were administered (Bupaq, Richter Pharma AG), and mice were kept in a warm environment during the recovery period. Secondary (F2) and tertiary (F3) transplantation: hematopoietic tissue from F1 PDX mice were applicated into sublethal irradiated mice (BM: intraosseal; LIV, SPL: intravenous) (procedure shown above).

### Bioluminescence-based engraftment monitoring

*In vivo* whole-body imaging of mice was performed every 4 weeks using the LagoX machine (Spectral Imaging Instruments) with Aura software. The mice were anaesthetized by 3% isoflurane inhalation and injected intraperitoneally with 300 µl of 15 mg/ml Xeno-light D-luciferine substrate (Perkin Elmer, 122799) to visualize the tumor cells. The luminescence exposure time was 300 s, and the X-ray image was taken subsequently. Data for the calculation of radiance were obtained from Spectral Instruments Imaging by Aura Imaging Software. Radiance is a calibrated absolute measurement of photon emission from the subject (photons/second/cm^2^/steradian). Mean Rad is defined as Total radiance/number of pixels in the ROI (region of interest) defined as the image area quantified.

### Flow cytometry analysis

Mouse blood samples (40-60 μl) were collected into EDTA-coated tubes. Red blood cells were lysed twice using ACK buffer. Following lysis, cells were washed with FACS buffer (2% FCS, 10 mM HEPES, 2 mM EDTA in HBSS without Ca^2+^ and Mg^2+^) and subsequently stained with a cocktail of fluorescently labeled antibodies supplemented with anti-mouse and human Fc block (Becton Dickinson 553142, 564220, both 1:200) in FACS buffer. The following antibodies were used: anti-hCD3-BV786 (Becton Dickinson 566781, 1:200), anti-hCD45-FITC (Biolegend 368508, 1:150), anti-hCD33-PECy7 (Biolegend 366618, 1:100), anti-hCD56-APC-Cy7 (Biolegend 362512, 1:200), and anti-mCD45-AF700 (Thermo Fisher Scientific 56-0451-82, 1:400). Following a washing step in FACS buffer, blood cells were resuspended in 195 μl of FACS buffer and mixed with 5 μl of CountBright™ Absolute Counting Beads (Thermo Fisher Scientific) and 0.5 μl of Hoechst 33258 (10 μg/ml). The entire sample was acquired on a Cytec Aurora cytometer. Data analysis was performed using FlowJo software, and absolute cell counts per 1 μl of blood were calculated.

### WST-1 assay and specific chemotherapeutic agents

The WST1 is a cell proliferation colorimetric assay (Roche, Basel, Switzerland) to obtain IC_50_ (concentration of drug required for 50% inhibition). WST-1 assay performed on primary AML samples, PDX samples and cell line OCI-M2-AZA-R. Cells were seeded (50 000 live cells/well) on 96 or 384-well flat bottom plate with freshly prepared inhibitors (concentration scale 1 nM – 10 µM; diluted in primary cells medium (RPMI1640 + 25% FBS + 10 ng/ml SCF, G-CSF, GM-CSF); total volume with cells 40 - 100 µL). Outer wells were filled with PBS to prevent evaporation. After 48 hours incubation period (in 37°C 5% CO_2_) WST-1 reagent was added to each well and absorbancy at 440 nm and 660 nm (reference value) was measured 60 min after addition using Tecan Reader. Each treatment condition was normalized to vehicle treatment and IC_50_ was calculated using Prism Graphpad software. Panel of specific inhibitors included: 5-Azacytidine (AZA), Venetoclax (VEN), Panobinostat (PAN), Dinaciclib (DIN) and Sorafenib (SOR). All inhibitors were purchased from MedChemExpress (Monmouth Junction, NJ, USA) or Selleckchem (Houston, TX, USA). These inhibitors were administered i.p. in human-equivalent dosages three times a week followed by a 7-day pause. AZA=2.14 mg/kg, VEN=5.7 mg/kg, PAN^high^ = 10 mg/kg, PAN^low^ = 5 mg/kg; DIN^high^ = 40 mg/kg, DIN^low^ = 20 mg/kg.

### Statistics

The data sets were compared using: t-test, Unpaired, two-tailed, confidence intervals 95%: *≤ 0.05, **≤ 0.001, ***≤ 0.0001, ****≤ 0.00001. Mean and error bars (SD, SEM) are shown.

## Results

### Investigating engraftment efficacy of PDX MDS/AML models

MDS/AML models can be derived from human leukemia cell lines (CDX models) or patients (PDX models) and are used to assess therapeutic efficacy and toxicity. Various models are currently available in commercial databases, but very little is known about how these models were generated and with what frequency. We set to determine whether the development of AML in PDX mice is a common or rare event. After xenotransplantation into irradiated NSGS recipients (as described in M&M and **Supplementary Figure 1A**), we monitored tumor tissue growth every two weeks by bioluminescence. We found that all 13 primary MDS/AML patient samples (**Table 1**) (using 34 NSGS mice, see **Supplementary Figure 1B**) were engrafted at 5 weeks after intraosseal injection. However, after 10 weeks, there was a sudden drop in increasing bioluminescence in some of the samples that led to complete loss of bioluminescent signal (i.e., 50% of the patient samples). This suggests that after a relatively long phase of proliferating engrafted tumor cells, either the proliferating capacity is diminished or external events such as tumor-specific lymphocytes may induce MDS/AML rejection.

**Table 1:** Characteristics of primary 13 MDS/AML patients for xenotransplantation into NSGS mice.

Sample	P6472	P6470	P6471	P6499	P7000	P7002	P7007	P7006	P7008	P6730	P6736	P6456	P6470 R
Sex	F	F	M	M	M	M	F	M	M	F	F	F	F
Age (y)	77	71	69	78	72	77	77	68	72	65	72	76	72
Sample Diagnosis (transformation)	AML M5	sAML (ET)	sAML (CMML)	AML M4	sAML (BPDCN)	sAML (MDS)	sAML (MDS)	AML M4	sAML (MDS)	AML M2	sAML (MDS)	sAML (MDS)	sAML (ET)
OS on VEN+AZA (mo)	4.47	16.23	7.8	1.7	5.8	4.03	6.5	15.13	25.3	12.63	8.43	16.23	16.23
2022 ELN risk class.	ADV	FAV	ADV	ADV	ADV	ADV	ADV	INT	INT	ADV	ADV	INT	ADV
BOR	NR	CR	NR	NR	NR	NR	NR	NR	NR	NR	CR	NR	NR
FISH	Neg	⚪	●	Neg	-7	del(7q)	del(5q)	◌	Neg	Neg	Complex	Neg	⚪
NGS	ASXL1 EZH2 TET2 NRAS	IDH2 NPM1	N/A	ASXL1 PTPN11 U2AF1	ASXL1 ZRSR2 CBL EZH2 TET2	IDH2 SRSF2 RUNX1 *EZH2	SF3B1 ASXL1	NRAS	N/A	ASXL1 BCOR NRAS RUNX1 SRSF2 STAG2	Tp53	NRAS	IDH2 NPM1 FLT3

ELN classification: ADV, Adverse; INT, Intermediate; FAV, Favorable.

Best Of Response (BOR): NR = No Response; CR Complete Remission.

FISH: ⚪ 46,XX, del(11)(q23.1q24.1) ● 46-47,XY,r(7)(p12q21.1),+21 ◌ 45,X,-Y,der(10)t(1;10)(q12;p13).

*loss of

R, Relapse.

In terms of tumor engraftment, the question is whether there is a repopulation of tumor-specific stem cells or whether the tumor growth in the first months consists only of short-lived leukemic cells, both of which are testable by the transplantation approach. To confirm the translatability of proliferating bioluminescent PDX-AML tumors, we performed a set of subsequent secondary and tertiary transplantations. Of the total number of transplanted samples, the number of those that were able to proliferate in secondary and tertiary recipients was reduced to 31% (i.e., 4 patient PDX models/out of 13 patients, **Supplementary Figure 1B**). These results suggest that the proliferative capacity of MDS/AML in the PDX model is exhausted between 5 and 10 weeks after transplantation, and approximately 1 out of 3 of MDS/AML samples retain repopulating stem-like activity.

### Molecular differences between MDS/AML sample and PDX model

**Figure 1:**
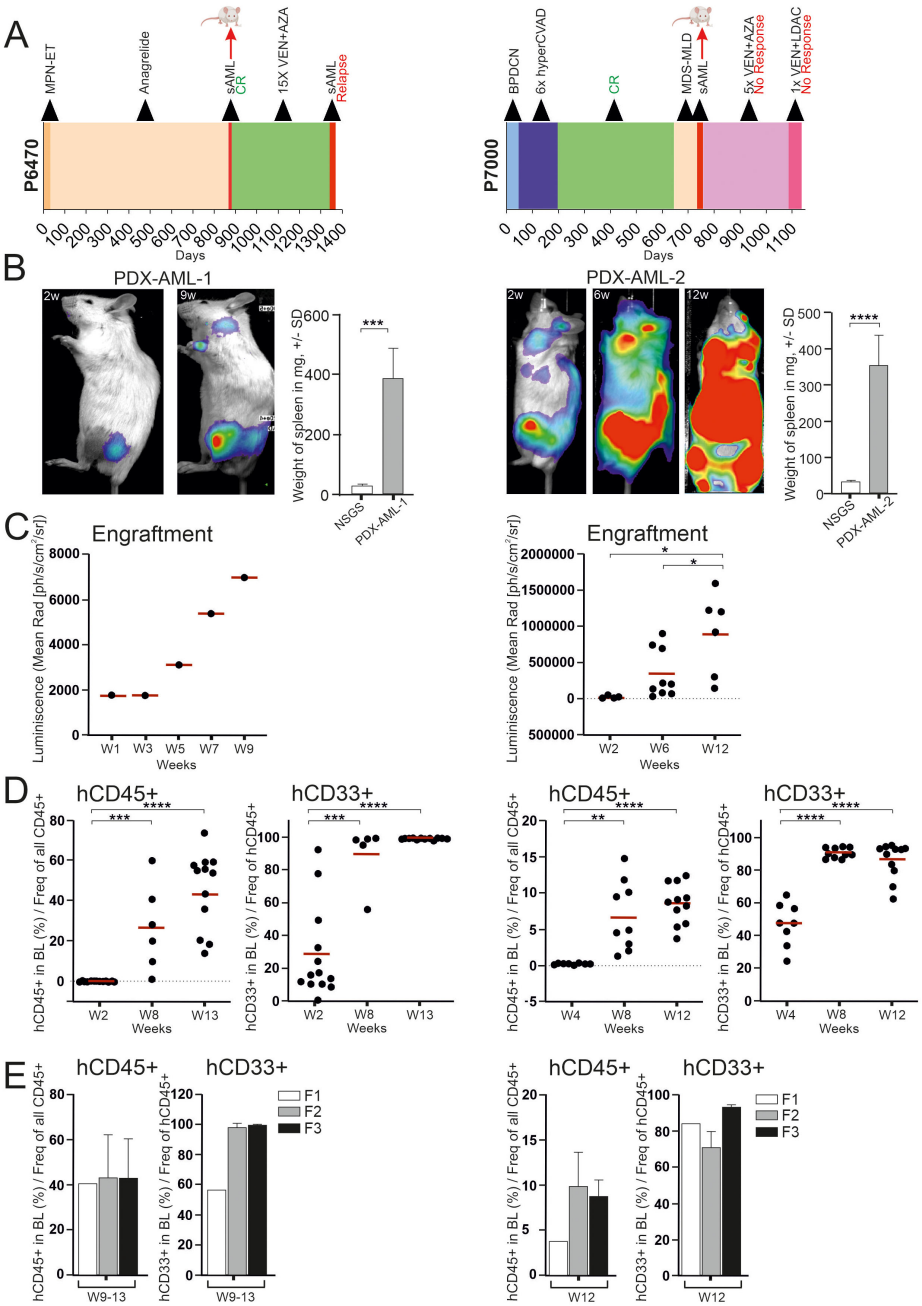
PDX model containing progressed MDS/AML with response to VEN (left side) and with resistance to VEN + AZA (right side). **(A)** Swimmer plots of two patients: P6470 with response to VEN (left) and P7000 with resistance to VEN + AZA (right). Graph includes a bar showing the length of treatment duration for each patient, the sample collection time for mouse transplantation is indicated by the red arrow. **(B)** Luminescence under general anesthesia (isoflurane) at indicated weeks after transplantation. Weight differences of NSGS spleens (N = 5) and PDX (N = 11, left; N = 8, right) in weeks 12-15. Mean ± SD, *p*-values (t-test, Unpaired, two-tailed). **(C)** Analysis of luciferase activity in PDX mouse (N = 1, during 9 weeks) (left) and in PDX mice at timepoints 2, 6, 12 weeks [W2-6 (N = 9), W12 (N = 6)] (right). Mean is shown, (t-test, Unpaired, two-tailed). **(D)** PB-FACS analysis of hCD45 or hCD33 (y-axis) at 2, 8 and 13 weeks (x-axis) [W2 (N = 13), W8 (N = 6), W13 (N = 12)] (left) and also PB-FACS analysis of indicated surface markers (y-axis) at 4, 8 and 12 weeks [W4 (N = 8), W8 (N = 10), W12 (N = 11)] (right). The data sets were compared using: t-test, Unpaired, two-tailed, confidence intervals 95%: *≤ 0.05, **≤ 0.001, ***≤ 0.0001, ****≤ 0.00001. Mean is shown. **(E)** PB-FACS analysis of indicated surface markers (y-axis) at terminal week in individual transplantation [F1 (N = 1), F2 (N = 8), F3 (N = 5)] (left) and [F1 (N = 1), F2 (N = 3), F3 (N = 7)] (right).

PDX models are widely used in research; their main advantage is that they are derived from patients, so they should faithfully recapitulate the context of the disease. On the other hand, it is unclear at this point whether a PDX model is completely identical to a patient sample. Therefore, we have taken a very detailed look at our generated PDX models using in depth methods (see **Figure 1**). All newly derived PDX models were assessed for tumor growth by bioluminescence and flow cytometry. Bioluminescence showed tumor growth from the primary site of inoculation (**Figure 1B**) and expansion of growth between weeks 1 and 9-12 of follow-up (**Figure 1C**). Analysis of the peritoneal cavity unveiled noticeable hepatosplenomegaly in PDX mice, which was notably more pronounced compared to the control NSGS mice (**Figure 1B**). Tumor analysis by flow cytometry showed accumulation of human CD45+ and CD33+ cells (**Figures 1D, E**). **Supplementary Figures 2A-B** show flow cytometry analysis after engraftment in blood and all hematopoietic tissue. **Supplementary Figure 2C** shows flow cytometry analysis after engraftment after the 1st, 2nd, and 3rd transplantation. Taken together, these data show that the newly created PDX models are unequivocally from leukemia stem cells, thus transplantable, and lead to significant tumor outgrowth in the recipients, resulting in overwhelming with tumor tissue.

**Figure 2:**
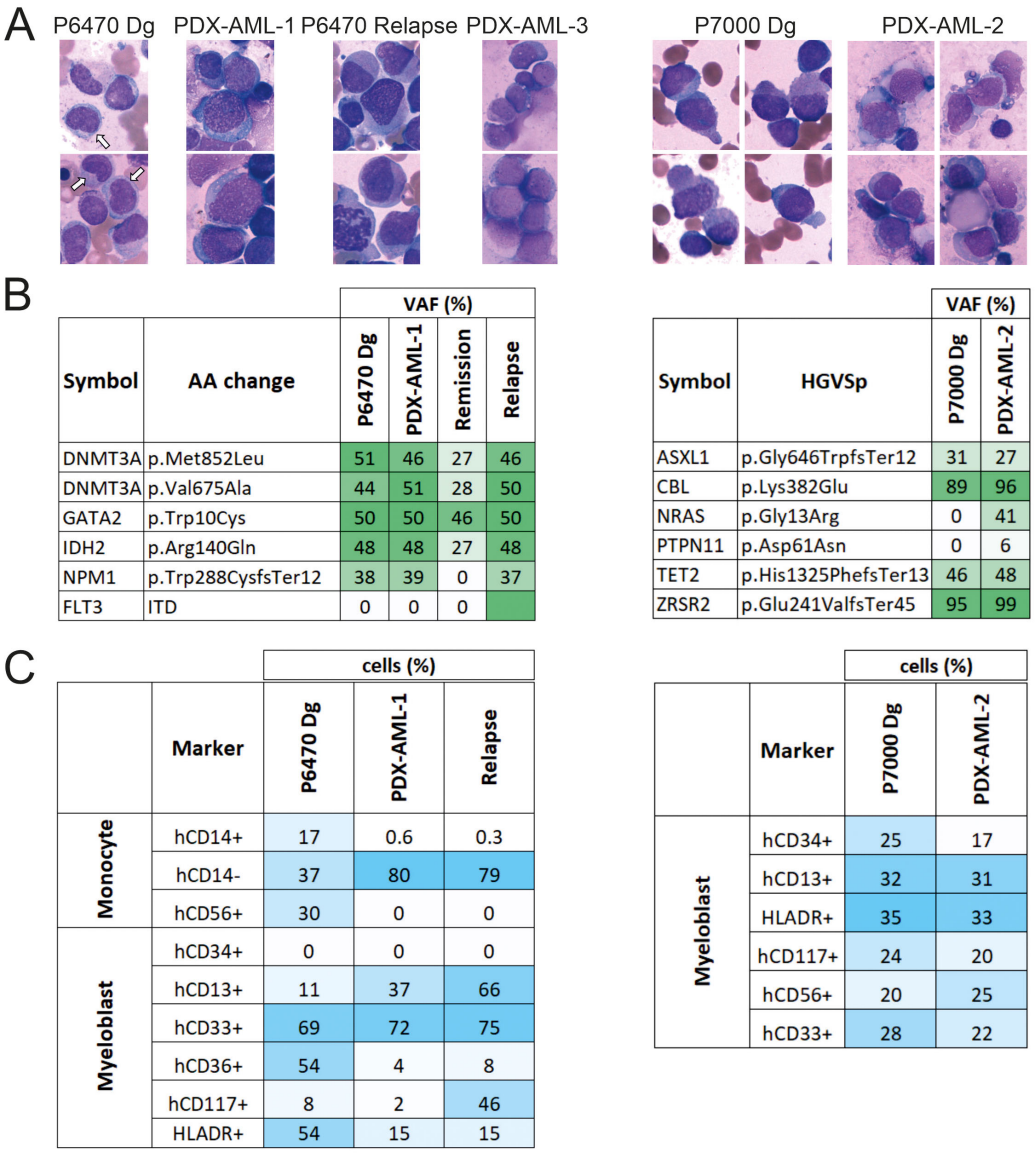
Comparison of the primary AML patient samples and the PDX-AML model. **(A)** Cytology of primary cells from diagnosis sample in comparison with cells from PDX-AML model stained via Giemsa-May-Grunwald protocol (white arrow = monocyte). **(B)** Mutation profile of primary samples in comparison with cells from PDX-AML models. **(C)** FACS analysis of indicated surface markers of primary samples in comparison with cells from PDX-AML model.

The PDX-AML-1 model was generated from a sample of female patient with secondary AML following ET who completed 15 cycles of VEN + AZA and achieved complete remission (CR) and progressed after 15 cycles of therapy (**Table 1**, **Figure 1A** left). NGS panel, flow cytometry and cytological analyses were used to compare tumor and PDX samples. Compared to the primary sample, the PDX-AML-1 model showed a loss of AML-associated monocytes (**Figure 2A**). The pathogenic mutations in *DNMT3A, GATA2* and *IDH2* persisted, however, unlike transient loss of *NPM1* bearing clone in the time of remission (**Figure 2B**). During the disease progression, we also observed the positivity of *FLT3-ITD*. FACS profile confirmed the loss of monocytes (**Figure 2C**; **Supplementary Figure 3A**). Interestingly, the flow cytometry results for PDX-AML-1 are very similar to those seen in this patient P6470 at relapse. Incidentally, the relapse of this patient was also transplanted into NSGS mice and successfully generated a PDX model, designated PDX-AML-3 (**Supplementary Figures 4A-D**; **Table 1** *in red*). Of note, the survival of the PDX-AML-3 mice is markedly shorter, which can be attributed to the selection of more aggressive clone/s (**Supplementary Figure 4D**).

The PDX-AML-2 model was derived from a male-patient with secondary/therapy-related AML following Blastic Plasmacytoid Dendritic Cell Neoplasm (BPDCN) who underwent 5 cycles of VEN + AZA achieving ‘No Response’ which was further managed with single cycle of VEN + *LowDose* AraC (**Table 1**, **Figure 1A** right); at this point the patient died due to robust progression and infection complication. Compared to the patient sample, the cytological (**Figure 2A**) and flow cytometry profiles (**Figure 2C**; **Supplementary Figure 3B**) were preserved in the PDX-AML-2 model, and pathogenic mutations of *ASXL1, CBL, TET2* and *ZRSR2* persisted alongside deletion of a chromosome 7. Importantly, additional mutations of *NRAS* and *PTPN11* were also found in the PDX sample (**Figure 2B**). Comparison of the primary sample with PDX MDS/AML shows that these are clearly very similar, but not identical, and that differences exist in cell surface markers that are formed or lost, cytological composition, and this is also reflected by the additional pathogenic mutations. These changes may arise from a limited number of stem cells that engraft MDS/AML within the mouse PDX. It was subsequently during the relapse that we noticed that the mutational profile of the PDX sample and the relapse showed some similarities, and the same can be said for the flow cytometry and cytology profile (see **Figures 2A-C**). Another PDX model designated as PDX-AML-4 was recently generated from patient P6730: 65-year-old women with AML M2 marked with mutations in *ASXL1, BCOR, NRAS, RUNX1, SRSF2*, and *STAG2* (see **Table 1** in red).

### Validating the therapeutic efficacy using PDX-derived cells and in a CDX model

**Figure 3:**
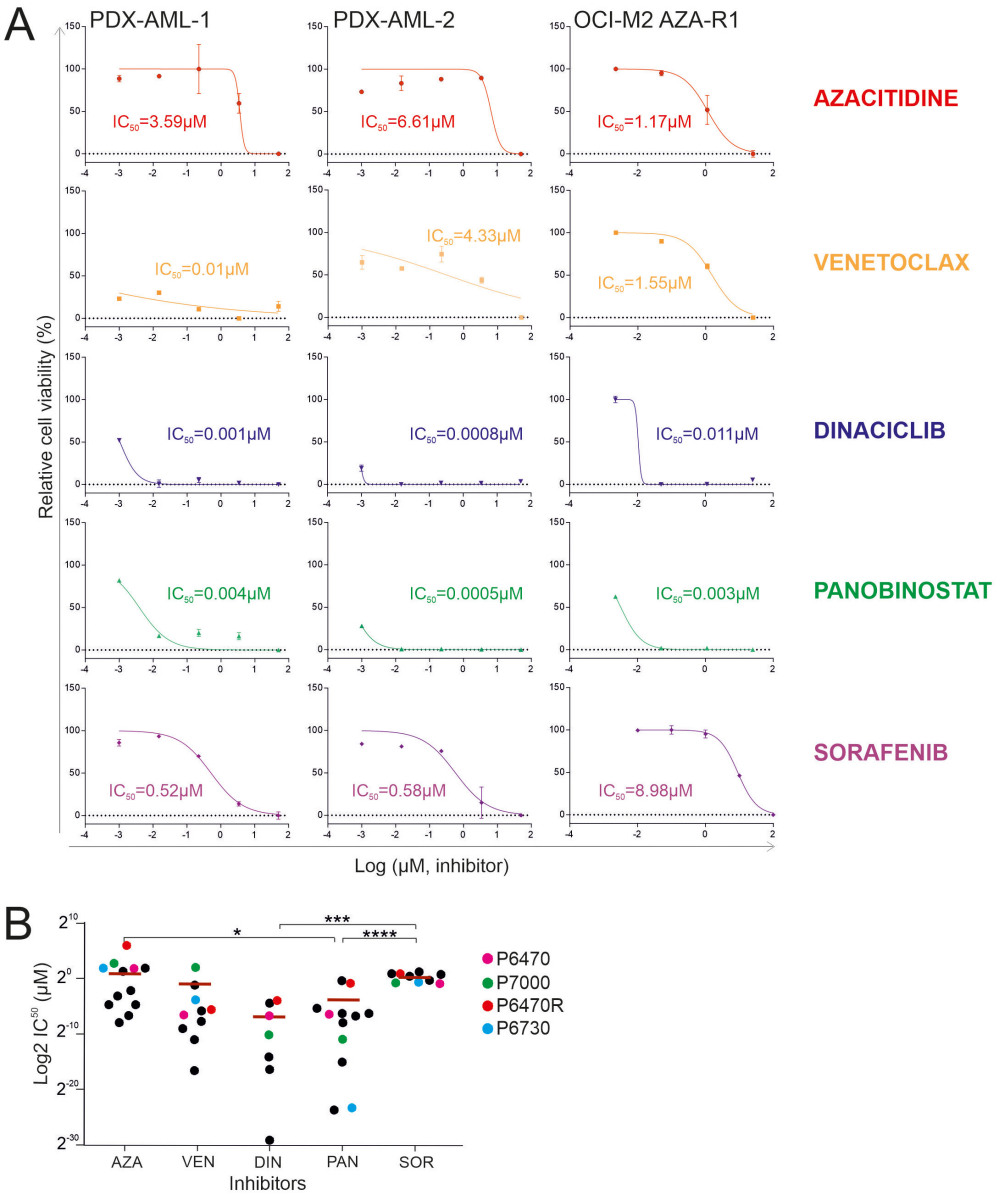
WST-1 proliferation essay. **(A)** Comparison of the effect of 5-Azacytidine (AZA), Venetoclax (VEN), Panobinostat (PAN), Dinaciclib (DIN) and Sorafenib (SOR) in primary MDS/AML cells and the AZA-resistant OCI-M2 cell line. **(B)** Comparison of IC50 values of following inhibitors: 5-Azacytidine (AZA), hypomethylating agent; Venetoclax (VEN), BCL2 inhibitor; Dinaciclib (DIN), CDK1/2/5/9 inhibitor; Panobinostat (PAN), HDAC inhibitor; Sorafenib (SOR) kinase inhibitor in AZA-resistant primary AML cells (N = 12). Data from established PDX models are shown in violet (P6470), red (P6470R), green (P7000), and blue (P6730). The data sets were compared using: t-test, Unpaired, two-tailed, confidence intervals 95%: *≤ 0.05, ***≤ 0.0001, ****≤ 0.00001. Mean is shown.

One way to assess the similarity or dissimilarity of a primary MDS/AML sample to PDX is to test for sensitivity to specific chemotherapeutic agents. Specifically, we validated resistance (seen in patient) to AZA (in the PDX-AML-1 model) and dual resistance to VEN + AZA (in the PDX-AML-2 model). To do so, we used the WST-1 assay in PDX-derived cells (**Figure 3A**). Our data show that the sensitivity to AZA and VEN in both PDX models is identical to the patient response to these drugs (**Figure 3B**). Considering this, we further tested other candidate agents. Specifically, we found shared resistance to Sorafenib (SOR) with which neither patient was treated. Conversely, we also found significant sensitivity to Dinaciclib (DIN; inhibitor of CDK 1/2/5/9) and Panobinostat (PAN; HDAC inhibitor). These results show that in the case of resistance to AZA or VEN + AZA, there is not an induction of multidrug resistance, but on the contrary, it seems that cells retained sensitivity to some other drugs, which can be used as a salvage option in case of resistance to AZA or AZA+VEN.

To further validate our assumptions derived from the experiment described in **Figure 3A**, which indicate the significant utility of Dinaciclib and Panobinostat for patients relapsing on the VEN + AZA regimen, we looked at the sensitivity of primary refractory progenitors following the VEN + AZA regimen to Dinaciclib and Panobinostat. Specifically, we used a cohort of patients treated with VEN + AZA. These patient samples (N=13) also include the samples that were used to successfully generate a PDX model. We used the WST1 method and measured the IC50 for the candidate drugs. We also further validated the IC50 for AZA and VEN. **Figure 3B** shows that Dinaciclib and Panobinostat are very effective alternatives for administration in patients with resistance to the VEN + AZA regimen, which cannot be stated for Sorafenib.

**Figure 4:**
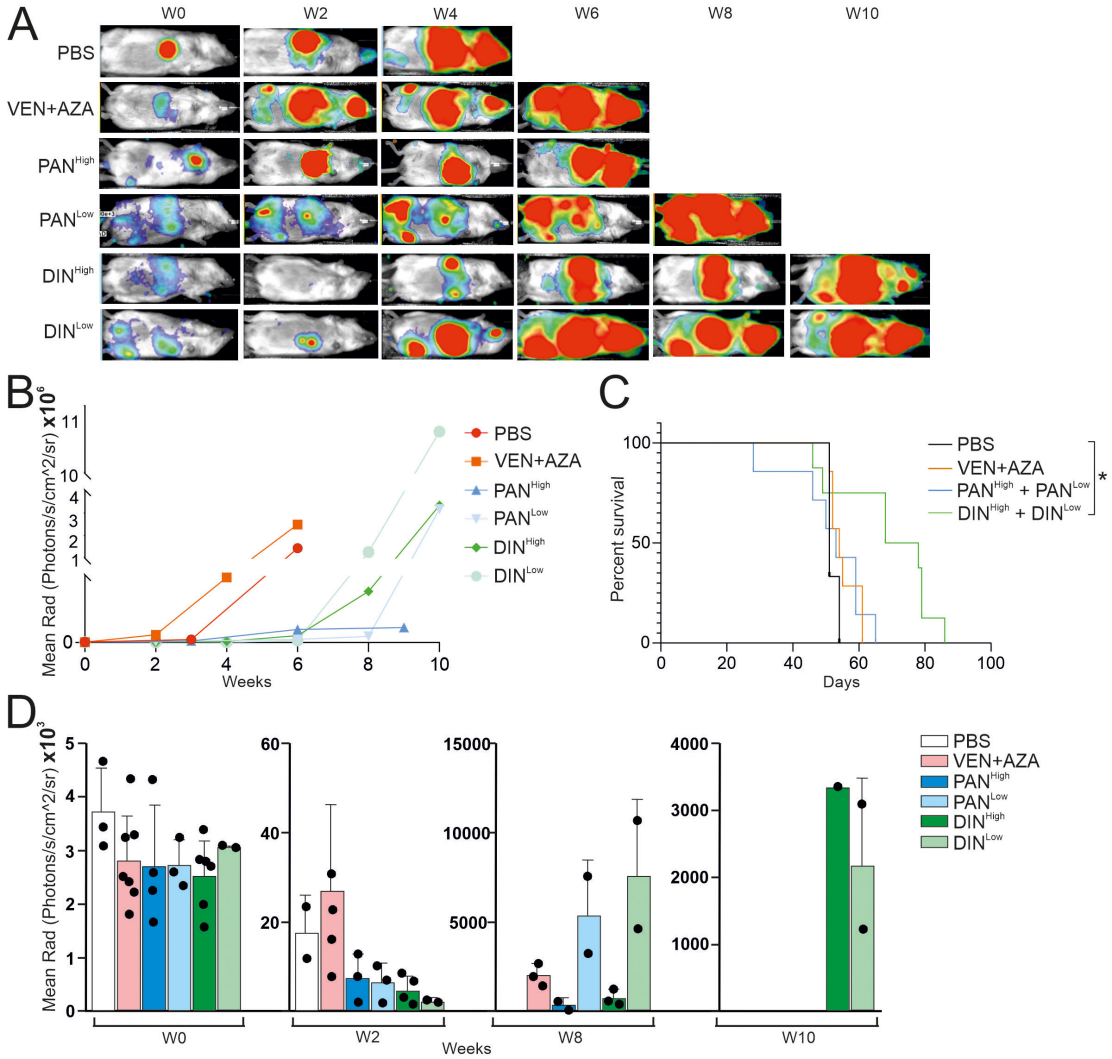
Testing the effect of PAN and DIN inhibitors on the survival of CDX mice containing the OCI-M2-AZA-R cell line. **(A)** Luminescence under general anesthesia (isoflurane) at indicated weeks after starting treatment with the inhibitors. **(B)** Analysis of luciferase activity in treated CDX mice (N=1 for each inhibitor treatment, during 10 weeks). **(C)** Survival curve of CDX treated mice [PBS (N=3), AZA/VEN (N=7), PAN^High^ (N=4), PAN^Low^ (N=3), DIN^High^ (N=6), DIN^Low^ (N=2)]. The data sets were compared using: t-test, Unpaired, two-tailed, confidence intervals 95%: *≤ 0.05. **(D)** Analysis of luciferase activity in treated CDX mice at 0, 2, 8 and 10 weeks after start of treatment.

To test the tumor-specific effect of PAN and DIN *in vivo*, we used CDX models that we have generated from the MDS/AML cell line OCI-M2 in the past and that are resistant to AZA and VEN. After i.v. injection of tumor cells, we first assessed whether the tumor cells had engrafted in the NSGS mice. Subsequently, after 2 weeks, we initiated therapy (placebo, VEN + AZA, PAN and DIN in two doses) and assessed their effect on tumor growth by bioluminescence. These data clearly showed that while control therapy (PBS) and treatment with VEN + AZA were unable to block rapid tumor accumulation in the mouse, tumor growth was more gradual in the case of PAN and DIN therapy (**Figure 4A**). Bioluminescence trends of the treated PDX models are shown in the **Figure 4B**. Tumor growth affects the survival of treated animals, specifically the DIN therapy (unlike of PAN) led to a significant prolongation of survival (**Figure 4C**). The evolution of bioluminescence over time (weeks 0, 2, 8, and 10) indicates that while all tumors have a roughly similar tumor signal at baseline, PAN and DIN signals are still visible at week 8 and only DIN signal is visible at week 10 (**Figure 4D**; **Supplementary Figure 5**). However, DIN appears to be relatively more effective to clear VEN + AZA resistant cells compared to PAN. Thus, in summary, we suggest that based on *in vitro* and *in vivo* modeling in CDX/PDX systems, novel and previously unconsidered drugs such as DIN or PAN can be considered for the treatment of VEN + AZA resistant MDS/AML patients in the future.

## Discussion

Our study showed that transplantable PDX models for MDS/AML resistant to VEN + AZA+ can be generated with a probability of 31% (~1 in 3) in NSGS mice. Lower engraftment efficiency as well as molecular signatures between PDX and primary AML have recently been also described, and it is more likely the leukemic initiating cells that determine this (7). In addition to the intrinsic nature of AML, there are also factors at the T cell level that can influence engraftment. Recently, a study of transgenic expression of interleukin IL15 improved T cell efficiency and thus survival in a PDX model. IL15 led to T cells remaining in a less differentiated state, thus preventing their exhaustion (15). In our PDX modeling, we also observed that in rare cases after loss AML engraftment, T cells accumulated at the engraftment site (data not shown), suggesting that the role of autologous lymphocytes is very important and possibly actionable. Previous studies have also shown a clonal discrepancy between PDX and primary cells. A recent study has shown that by investigating the clonal dynamics of somatic mutations in the PDX system, Resistant/Refractory (R/R) AMLs dominate in PDX mice generated from multi-clonal AML cells at diagnosis, although R/R clones have low allelic frequency in a diagnosis sample, indicating their engraftment potential (16).

Our study showed that resistance to VEN + AZA can be overcome with some already FDA approved drugs. Specifically, DIN and to lesser extent also PAN are two effective inhibitors that can be considered in patients with developed VEN + AZA resistance who are at risk of very short survival upon VEN + AZA failure. However, clinical validation of this preclinical result remains one of the long-term goals resulting from this work. Although several mechanisms of VEN resistance in AML have been identified, recent research has shown that heterogeneity in resistance mechanisms across patient populations exists also at the transcriptomic and cell signaling levels, specifically with activation of the PI3K-AKT-mTOR signaling axis and energy metabolism pathways. Other transcriptional features included transcriptional repression of HOX expression, activation of JAK-STAT signaling, or overexpression of interferon signaling (17). Given the significant transcriptional differences in VEN resistance, the efficacy of HDAC inhibitors is understandable. Similar and detailed results were also reported by another study, which investigated the mechanisms by which the anti-AML activity of VEN could be potentiated by dual mTORC1/TORC2 inhibition. Among others, for example, constitutive AKT activation counteracted the synergism between Venetoclax and PI3K or AKT inhibitors (18). We previously showed for the CDX AZA-resistant model (based on the OCI-M2 MDS/AML cell line) that AKT signaling plays an important and actionable role (19). Recent research has shown that a VEN in combination with the PPARα agonist chiglitazar led to synergistic suppression of AML progression in PDX models. Mechanistically, chiglitazar-mediated PPARα activation inhibited the transcriptional activity of the PIK3AP1 gene promoter and down-regulated the PI3K/AKT signaling pathway and anti-apoptotic BCL2 proteins, leading to inhibition of cell proliferation and induction of apoptosis, which was synergized with VEN (20). Another work suggested another unique mechanism that via AXL/MERTK inhibition effectively blocks VEN-resistant FLT3-ITD AML cells including those overexpressing MCL1 (21). Another recent study showed that increased expression of the ubiquitin ligase RNF5 contributes to the development and survival of AML, while inhibition of RNF5 causes transcriptional changes that overlap with those observed with histone deacetylase (HDAC) inhibition. RNF5 induces the formation of K29 ubiquitin chains on the histone-binding protein RBBP4, thereby promoting its recruitment and subsequent epigenetic regulation of genes involved in AML maintenance. Therefore, knockdown of RNF5 or RBBP4 increases the sensitivity of AML cells to HDAC inhibitors (22). Considering these results, it is likely that susceptibility to HDAC inhibitors in AML may be potentiated or may arise because of clonal selection during development of VEN + AZA resistance.

Like what we have attempted, there are laboratories developing new AML PDX models (23). There are even already PDX models with a particular pattern of mutations, specifically e.g. DNMT3A mutants, on which specific therapies can be tested (24). In addition, a methodological article to support the development of new PDX models has recently also been published (25). PDX research has led to the introduction of new therapies, specifically some agents such as CD47 inhibitors can sensitize to VEN + AZA treatment (26). The stress sensor GADD45A in AML is also associated with treatment resistance in AML and PDX models have been utilized during this research (27). PDX models have also been used to understand the role of “multiple drug resistance” mechanisms in AML (28) or to test novel CAR-T therapies (29). Thus, the role of PDX models in modern AML pharmacotherapy is beyond dispute and in this respect our work represents a step towards testing new approaches to VEN + AZA resistance and introducing new therapies.

## Data Availability

The original contributions presented in the study are included in the article/**Supplementary Material**. Further inquiries can be directed to the corresponding author.
