# Supplementary material for: Investigating resistance to 5-Azacytidine and Venetoclax in PDX models of MDS/AML
Source: Front Oncol. 2025 Jan 7;14:1414950. doi: 10.3389/fonc.2024.1414950 (PMC11747314; doi:10.3389/fonc.2024.1414950)
Supplement: Supplementary file 1 [file DataSheet1.docx]

**Supplementary information for:**

**Investigating Resistance to 5-Azacytidine and Venetoclax in PDX Models of MDS/AML.**

Petra Basova^1†^ and Lubomir Minarik^1,2†^, Silvia Carina Magalhaes Novais^3^, Jana Balounová^3^, Zuzana Zemanová^4^, Tatiana Aghová^4^, Martin Špaček^4^, Anna Jonasova^2^, Kristýna Gloc Pimková^1^, Jan Procházka^3^, Radislav Sedlacek^3^, Tomas Stopka^1,2*^

^†^ These authors contributed equally to this work and share first authorship.

**Supplementary figures:**

**Supplementary Figure 1A**


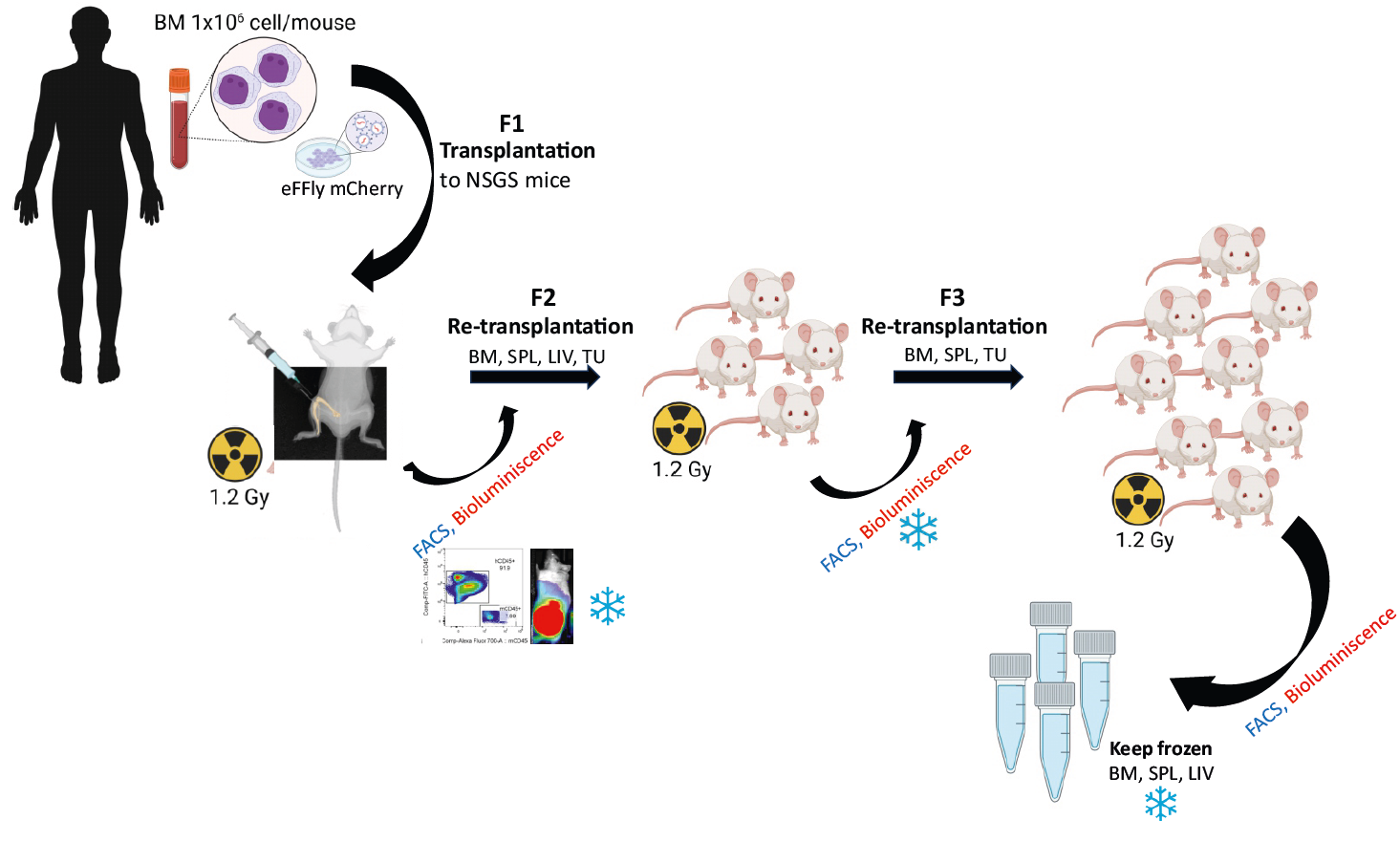


**Supplement Figure 1A.** **Scheme of the preparation of the new PDX model.** **(A)** High-risk MDS/AML BM cells (1x10^6^ mCherry labeled cells/mouse) (donor) were transplanted intraosseously (F1) into sublethally irradiated NSGS recipients. Bioluminescence intensity and blood surface markers hCD45+, hCD33+ were measured. After engraftment, tissues (bone marrow (BM), spleen (SPL), liver (LIV) and tumour (TU)) were harvested and used for further re-transplantation (F2, i.o.) into sublethally irradiated NSGS recipients. After engraftment, further re-transplantation (F3, i.o.) into sublethally irradiated NSGS recipients followed. Engrafted patient tumor cells were harvested and cryopreserved for further drug screening assays.

**Supplementary Figure 1B**

**
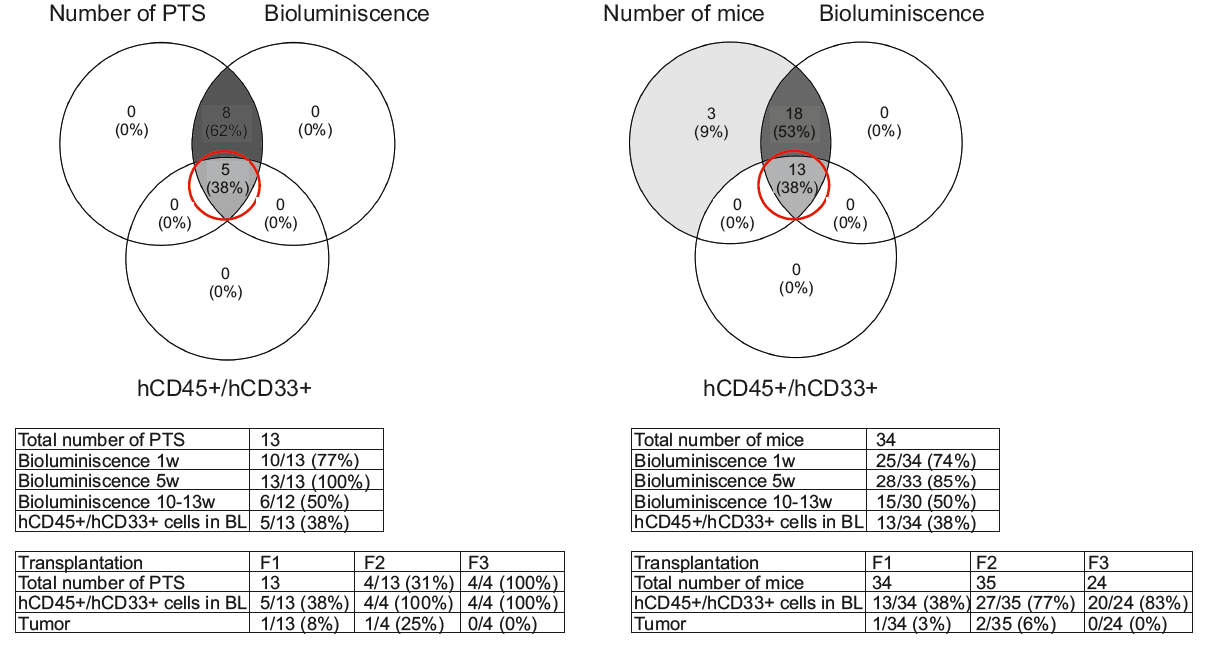
**

**Supplement Figure 1B. Venn diagrams.** The diagram on the left shows the number of MDS/AML patients (N = 13). On the right is a diagram with the number of NSGS mice used for transplantation (N = 34). The tables below the diagrams indicate the percentage of engraftment success at each period (1w, 5w and terminal) of transplantation.


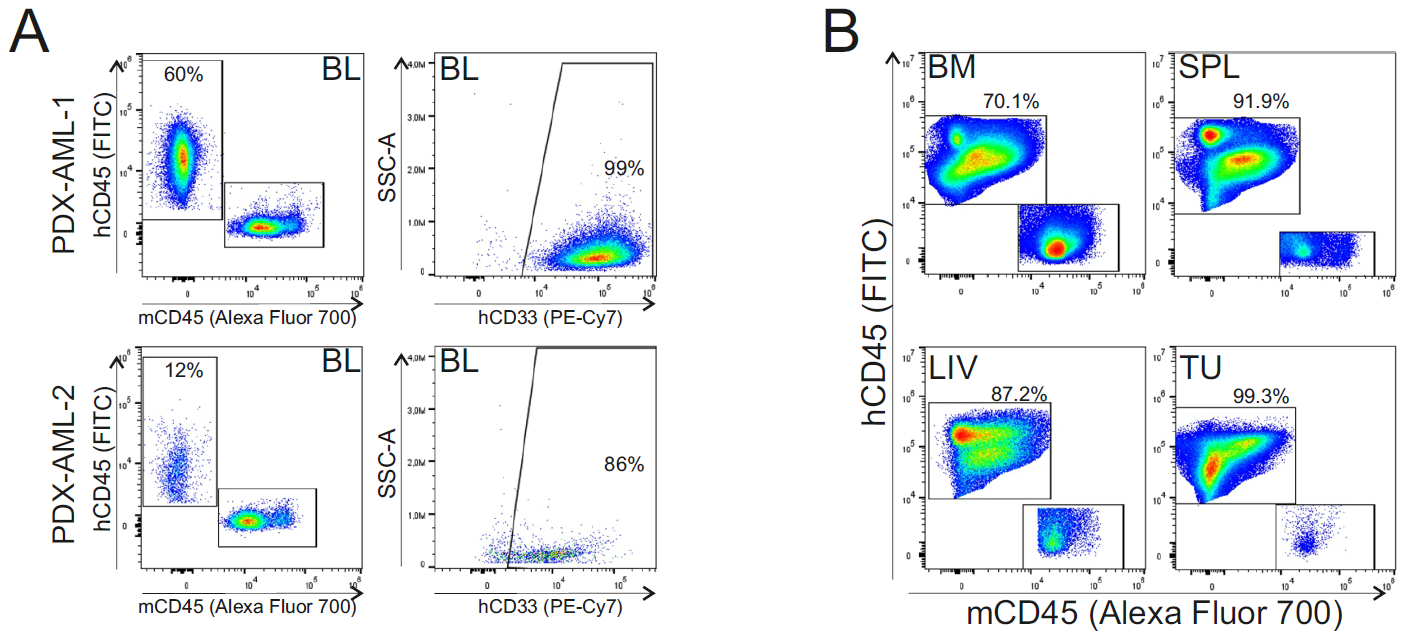
**Supplementary Figures 2A and 2B**

**Supplement Figures 2A and 2B. FACS analysis of PDX models. (A) (Left)** PB-FACS analysis of hCD45 (FITC) (y-axis) and mCD45 (Alexa Fluor 7000) (x-axis). **(Right)** PB-FACS analysis of hCD33 (PE-Cy7) (x axis). Quadrant display % of positive cells. hCD33^+^ cells represent immature AML phenotype. **(B)** Flow cytometry for hCD45 (FITC) (y-axis) and mCD45 (Alexa Fluor 7000) (x-axis) in BM, spleen, liver and tumor. Quadrants display % of positive cells.

**Supplementary Figures 2C**

**
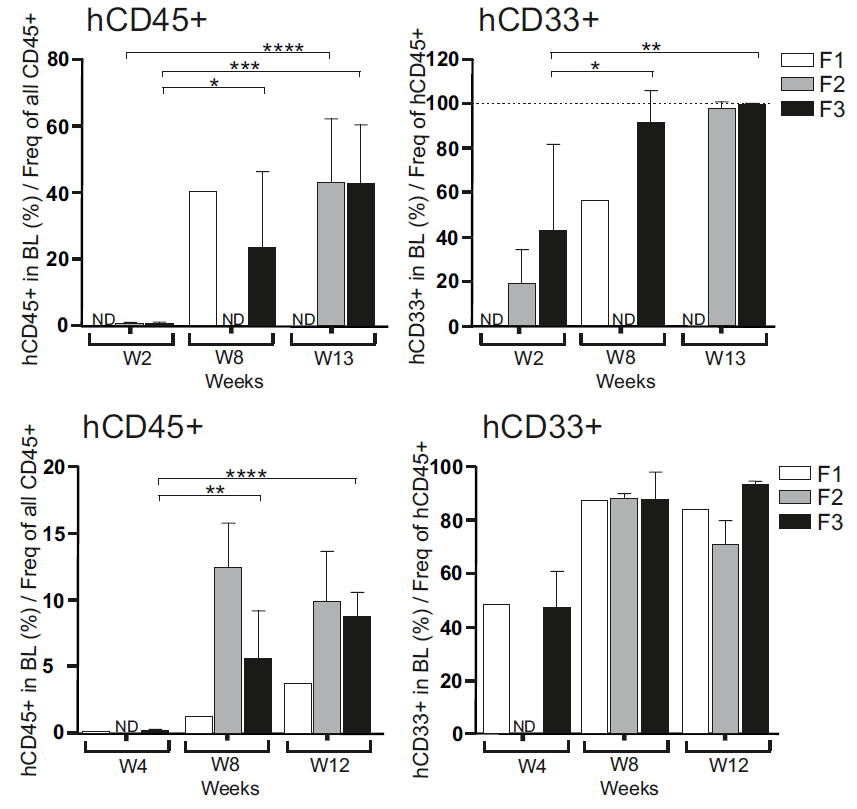
**

**Supplementary Figures 2C. FACS analysis of PDX models. (Up)** PDX-AML-1: PB-FACS analysis of indicated surface markers (y-axis) at 2, 8 and 13 weeks in individual transplantation (F1 (N = 1), F2 (N = 8), F3 (N = 5)). **(Down)** PDX-AML-2: PB-FACS analysis of indicated surface markers (y-axis) at 4, 8 and 12 weeks in individual transplantation (F1 (N = 1), F2 (N = 3), F3 (N = 7)). *p*-values: *≤ 0.05, **≤ 0.001, ***≤ 0.0001, ****≤ 0.00001.

**Supplementary Figures 3A**

**
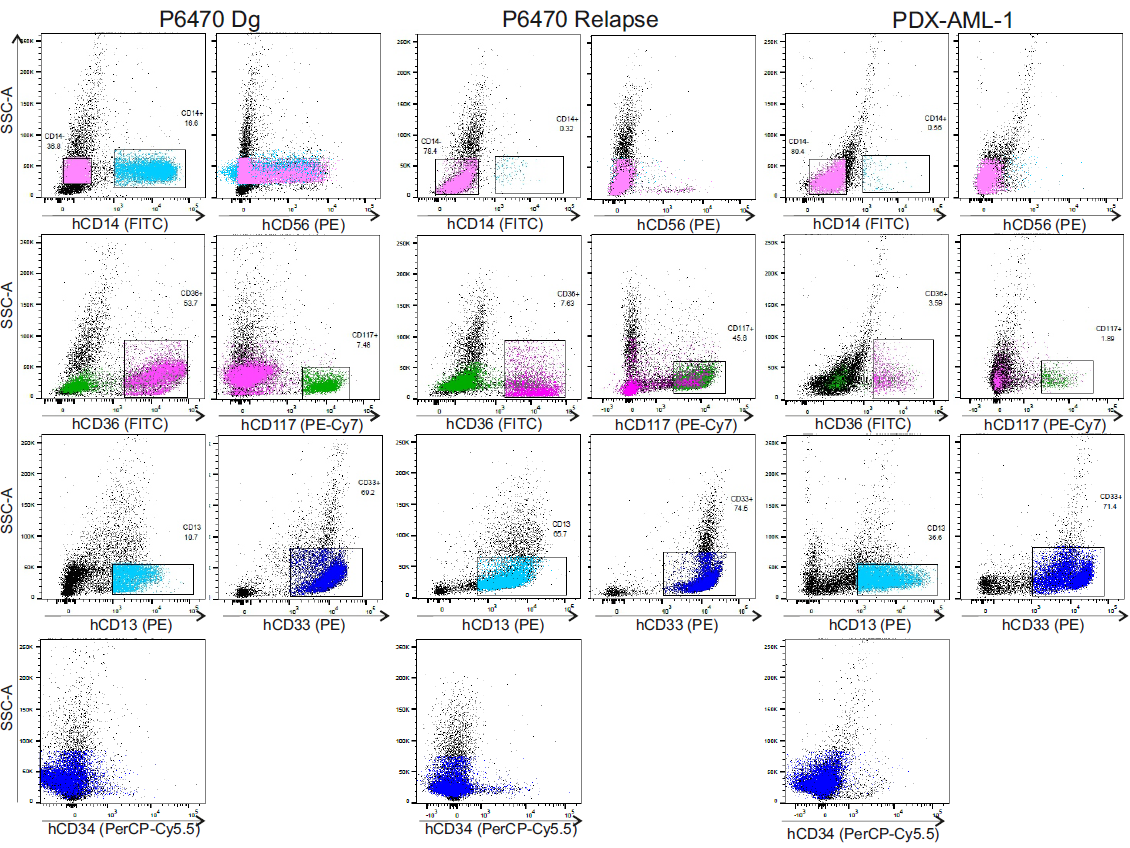
**

**Supplement Figure 3A. BM-FACS analysis of primary samples in comparison with cells from PDX-AML-1 model.** FACS analysis of indicated surface markers (x-axis) and SSC-A (y-axis).

**Supplementary Figures 3B**

**
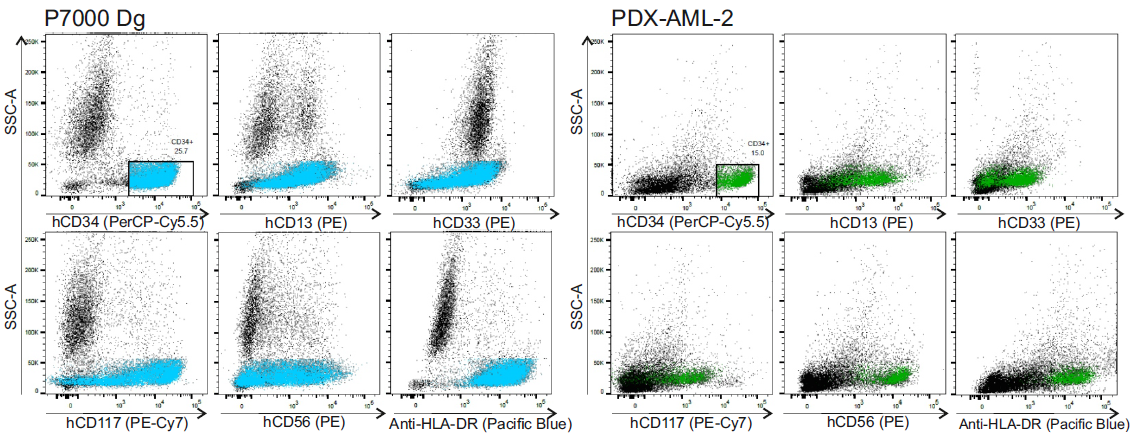
Supplement Figure 3B. BM-FACS analysis of primary samples in comparison with cells from PDX-AML-2 model.** FACS analysis of indicated surface markers (x-axis) and SSC-A (y-axis).

**Supplementary Figures 4A and 4B.**

**
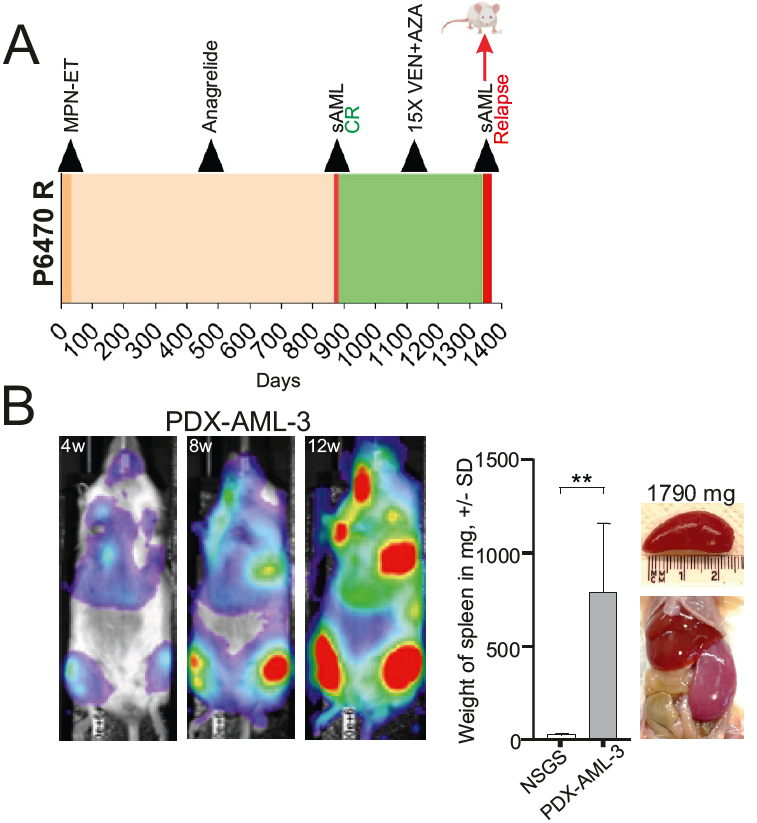
**

**Supplement Figure 4A and 4B. Developement of the VEN + AZA resistant PDX-AML-3 model**. (**A)** Clinical course of the female patient diagnosed with secondary AML transformed from ET. She achieved CR on VEN + AZA therapy, followed by the relapse driven by expansion of subclone bearing *FLT3*-ITD. The sample P6470R was obtained from the relapse and is labelled as the red arrow. X-axis shows the treatment duration in days. (**B**) Bioluminiscence of luciferase labelled cells is shown. Measurements were performed every 4 weeks. Comparison of weights of spleens in control mice (NSGS, N = 5) vs. engrafted mice (PDX-AML-3, N=12). Mean ± SD, *p*-values: **≤ 0.001 (t-test, Unpaired, two-tailed).

**Supplementary Figures 4C and 4D**

**
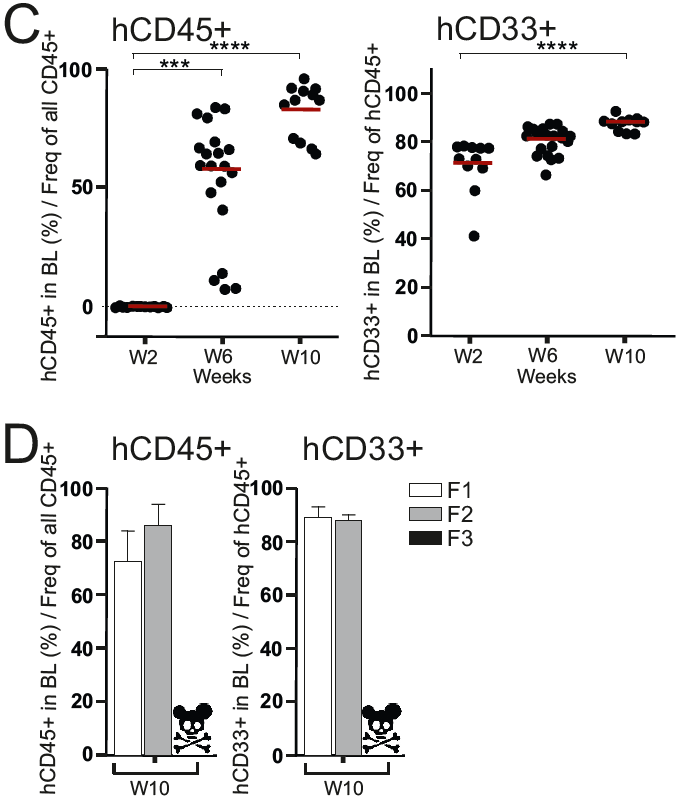
**

**Supplement Figure 4C and 4D. PDX-AML-3: flow cytometry analysis. (C)** PB-FACS analysis of hCD45 or hCD33 (y-axis) at 2, 6 and 10 weeks (x-axis) (W2 (N = 11), W6 (N = 20), W10 (N = 12)). The data sets were compared using: t-test, Unpaired, two-tailed, confidence intervals 95%: ***≤ 0.0001, ****≤ 0.00001. Mean is shown. **(D)** PB-FACS analysis of indicated surface markers (y-axis) at week 10 (at the time experiment termination) in each of the three engraftments (F1 (N = 4), F2 (N = 8), F3 (note that overall survival of the F3 mice was less than 8 weeks)).

**Supplementary Figure 5**


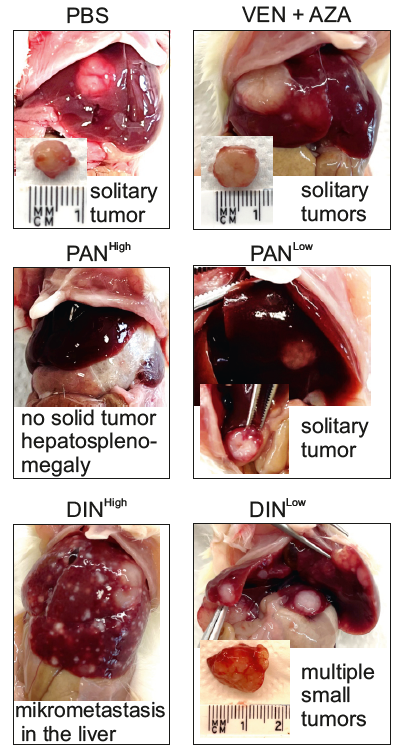


**Supplement Figure 5: Macroscopic examination of the CDX mice engrafyed with human AML cell line OCI-M2.**
